# Incidence and Characteristics of Scarlet Fever, South Korea, 2008–2015

**DOI:** 10.3201/eid2304.160773

**Published:** 2017-04

**Authors:** Duck Woong Park, Sun-Hee Kim, Jung Wook Park, Min-Ji Kim, Sun Ju Cho, Hye Jung Park, So Hyang Jung, Mi Hee Seo, Yong Seok Lee, Byung Hee Kim, Hyeran Min, Su Ya Lee, Dong Ryong Ha, Eun Sun Kim, Yeongjin Hong, Jae Keun Chung

**Affiliations:** Health and Environment Research Institute of Gwangju, Gwangju, South Korea (D.W. Park, S.-H. Kim, J.W. Park, M.-J. Kim, S.J. Cho, H.J. Park, S.H. Jung, M.H. Seo, Y.S. Lee, D.R. Ha, E.S. Kim, J.K. Chung);; Mirae Children’s Hospital (NamGu), Gwangju (B.H. Kim);; Jungang Children’s Hospital, Gwangju (H. Min);; Yesarang Pediatric Clinic, Gwangju (S.Y. Lee);; Chonnam National University Medical School, Gwangju (D.W. Park, Y. Hong)

**Keywords:** scarlet fever, *emm*, exotoxin, disk diffusion antimicrobial tests, South Korea, group A strep, bacteria, streptococci, virulence factors, antimicrobial resistance, *Streptococcus pyogenes*

## Abstract

The incidence rate for scarlet fever in South Korea is rising. During 2008–2015, we collected group A *Streptococcus* isolates and performed *emm* and exotoxin genotyping and disk-diffusion antimicrobial tests. Scarlet fever in South Korea was most closely associated with *emm* types *emm*4, *emm*28, *emm*1, and *emm*3. In 2015, tetracycline resistance started increasing.

Scarlet fever is a common disease caused by group A *Streptococcus* (GAS; also known as *Streptococcus pyogenes*). In the Far East and the United Kingdom, the incidence of scarlet fever has been increasing since 2008 (*1*–*3*), and according to the Infectious Disease Statistics System of Korea, the incidence rate for scarlet fever in South Korea increased from 0.3 cases/100,000 persons in 2008 to 13.7 cases/100,000 persons in 2015 (https://is.cdc.go.kr/dstat/index.jsp).

Several antimicrobial drugs, including β-lactams and tetracyclines, effectively treat scarlet fever, and macrolides and lincosamides can be used in patients with penicillin (β-lactam) allergy (*4*,*5*). However, resistance to erythromycin and clindamycin has been reported for GAS isolates in mainland China and Hong Kong, China (*2*,*3*). The streptococcal M protein and exotoxins are 2 of several virulence factors in GAS (*6*). The streptococcal M protein is a long fimbrial adhesion protein encoded by >220 M protein gene sequence types (*emm* types). Because of the high genetic variability of *emm*, which varies by geographic region, molecular *emm* genotyping is mandatory for epidemiologic investigations of GAS infections (*7*). The incidence of these infections is closely related to variations in the predominance of certain *emm* types (*7*). *spe*A and *spe*C, which are 2 of 11 genes encoding for superantigens found in GAS, are often associated with scarlet fever (*8*). Our objective was to identify the overall trend in the annual incidence and characteristics of scarlet fever in South Korea by studying its upsurge in Gwangju, South Korea, because incidence during the past 8 years was highest for this city (https://is.cdc.go.kr/dstat/index.jsp).

## The Study

**Figure 1:**
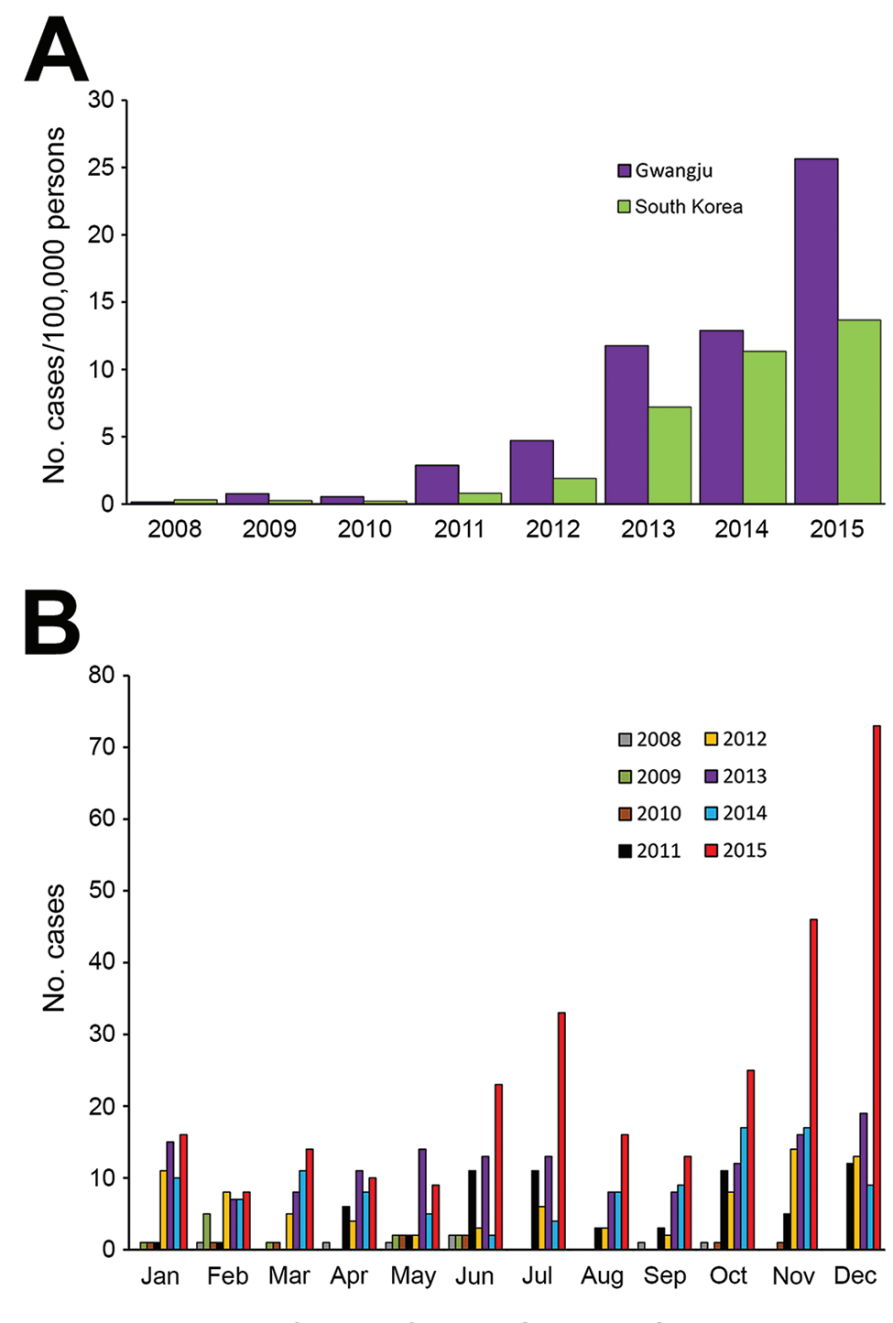
Incidence of scarlet fever in Gwangju, South Korea, 2008–2015. A) The number of cases per 100,000 persons in Gwangju and South Korea. B) Distribution of cases by month of each year.

The incidence of scarlet fever in the Gwangju metropolitan area is the highest among all South Korea cities (61.5 cases/100,000 persons); according to the Korean Disease Web Statistics System, the national incidence from 2008 through 2015 was 36.9 cases/100,000 persons (https://is.cdc.go.kr/dstat/index.jsp). Incidence of scarlet fever in South Korea began to increase in 2011 (Figure 1, panel A), coinciding with an outbreak of scarlet fever in China and Hong Kong. Scarlet fever mainly occurs during the late fall, winter, and early spring. Our study results indicate that the incidence of scarlet fever in South Korea peaks in the winter; however, it also peaked in the summers of 2011 and 2015 (Figure 1, panel B).

During 2008­–2015, we collected 1,460 pharyngeal swab samples from patients suspected of having scarlet fever from 8 major hospitals in the Gwangju metropolitan area. We tested the β-hemolytic isolates for susceptibility to bacitracin (0.04 U) and for streptococcal grouping; a total of 705 samples were positive for GAS.

**Figure 2:**
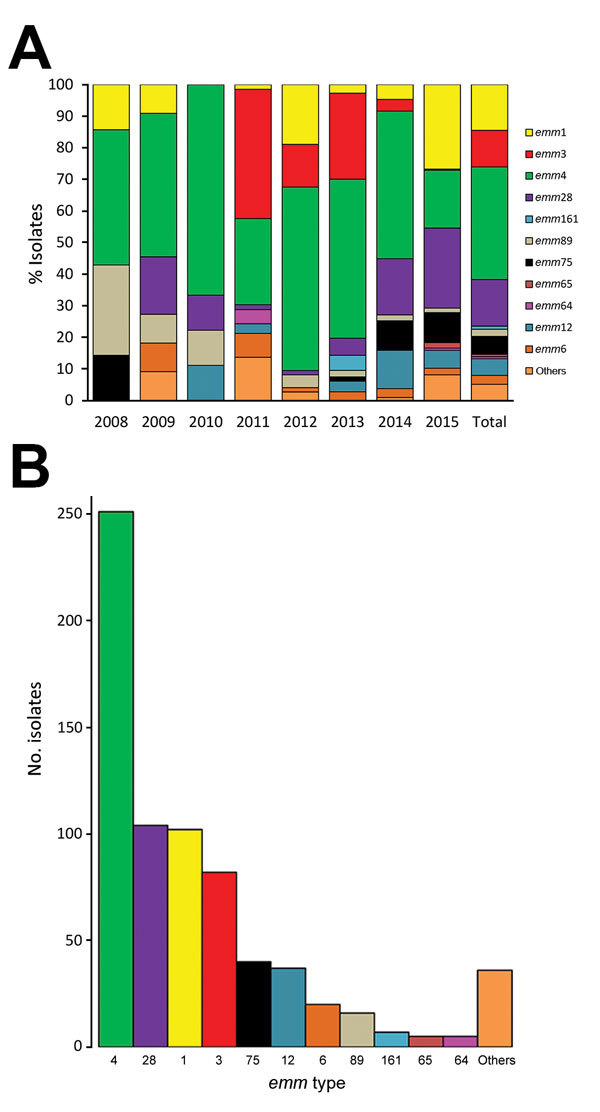
*emm* type characterization of group A *Streptococcus* isolates from patients with scarlet fever, Gwangju, South Korea, 2008–2015. A) Annual fluctuations of *emm* types. Number of isolates by year: 7 in 2008, 11 in 2009, 9 in 2010, 66 in 2011, 74 in 2012, 147 in 2013, 107 in 2014, and 284 in 2015. B) Total number of isolates by *emm* type. Others refers to rarely found *emm* types (*emm*11, *emm*13, *emm*17, *emm*23, *emm*26, *emm*30, *emm*31, *emm*43, *emm*49, *emm*59, *emm*81, *emm*82, *emm*87, *emm*101, *emm*107, *emm*131, *emm*135, *emm*161, *emm*163, *emm*174, *emm*183, *emm*196, *emm*203, *emm*204, *emm*227, *emm*236, and *emm*241).

Because variation in the circulating *emm* types could contribute to the incidence of disease and changes in the epidemiology of scarlet fever, we determined the *emm* sequence type for the 705 samples by using a standard protocol (https://www.cdc.gov/streplab/protocol-emm-type.html) (*2*). A total of 11 different *emm* sequence types were identified (Figure 2, panels A and B). *emm*4 (35.6%, 251/705) was the most predominant. The other 3 predominant *emm* types were *emm*28 (14.8%, 104/705), *emm*1 (14.5%, 102/705), and *emm*3 (11.6%, 82/705), and these 4 *emm* types accounted for ≈76.5% (539/705) of all isolates. These results differed from those of previous studies in mainland China and Hong Kong, where the outbreaks of scarlet fever were caused mainly by *emm*12 (*2*,*3*). Our results showed that *emm*3 in 2011 (the year incidence began increasing in South Korea) and *emm*1 and *emm*28 in 2015 (the year of a sharp increase in incidence) played major roles in the epidemics in the Gwangju metropolitan area (Figure 2, panel A). To our knowledge, *emm*3 has not been reported to be prevalent in other Asian countries but has been associated with scarlet fever in the United Kingdom (*9*).

Scarlet fever is a toxin-mediated disease (*10*). Therefore, we tested all isolates for the presence of the *spe*A and *spe*C genes by using PCR and previously reported primer pairs and reaction conditions (*8*). We found isolates that harbored *spe*A (57/705, 8.1%), *speC* (249/705, 35.3%), and both (3/705, 0.4%). The exotoxin gene detection rate differed by *emm* gene type. The isolates positive for *spe*A were predominantly *emm*1 (56.1%, 32/57) and *emm*28 (28.1%, 16/57); the other *emm* types made up only 15.8% (9/57). When we examined the reverse association, 31.4% (32/102) of *emm*1 isolates and 15.4% (16/104) of *emm*28 isolates were positive for *spe*A; in contrast, only 1.8% (9/499) of the other *emm* types were positive for *spe*A. The main *emm* types identified in the *spe*C-positive isolates were *emm*4 (68.7%, 171/249), *emm*75 (10.8%, 27/249), and *emm*28 (6.4%, 16/249); 68.1% (171/251) of *emm*4, 67.5% (27/40) of *emm*75, and 15.4% (16/104) of *emm*28 isolates were positive for *spe*C. Only 11.3% (35/310) of the other *emm* types were positive for *spe*C. Therefore, *spe*A and *spe*C exotoxin genes were more prevalent in bacteria of certain *emm* types (p<0.01).

GAS remains universally susceptible to β-lactams and glycopeptides. However, the rates of resistance against the macrolides and lincosamides used in penicillin-allergic patients have increased (*11*). We performed susceptibility tests by using the disk-diffusion method as recommended by the Clinical and Laboratory Standards Institute (*12*). For all samples collected 2008–2015, the antimicrobial agents chloramphenicol, tetracycline, erythromycin, and clindamycin were tested. Resistance to antimicrobial drugs was detected in 9.1% (64/705) of isolates: 0.3% of the isolates (2/705) showed resistance to chloramphenicol, 7.0% (49/705) to tetracycline, 3.0% (21/705) to erythromycin, and 2.8% (20/705) to clindamycin (Table).

**Table:** Characterization of antimicrobial drug resistance according to *emm* types in Gwangju, South Korea, 2008–2015*

Year, antimicrobial drug	*emm* type					
	*emm*1	*emm*4	*emm*12	*emm*28	Others	Total
2008						
Tetracycline	–	1†	–	–	–	1†
2009						
Erythromycin	–	–	–	2	–	2
Clindamycin	–	–	–	2	–	2
Tetracycline	–	–	–	1	–	1
2010						
Erythromycin	–	–	–	1	–	1
Clindamycin	–	–	–	1	–	1
2011						
Erythromycin	–	1	–	1	1†	1/3†
Clindamycin	–	1	–	1	–	2
Tetracycline	–	–	–	1	–	1
2012						
Erythromycin	–	–	–	1	–	1
Clindamycin	–	1	–	1	–	2
Tetracycline	–	1	–	–	–	1
2013						
Chloramphenicol	1	–	–	–	–	1
Erythromycin	1	–	–	2	1†	1/4†
Clindamycin	–	1	–	2	–	3
Tetracycline	–	1	–	1	–	2
2014						
Chloramphenicol	–	–	1†	–	–	1†
Erythromycin	–	–	–	4	–	4
Clindamycin	–	–	–	4	–	4
Tetracycline	–	1†	–	4	–	1/5†
2015						
Erythromycin	–	–	1	5	–	6
Clindamycin	–	–	1	5	–	6
Tetracycline	22/23†	–	1	2/7†	5/7†	29/38†
Isolates, % (no./total)	24.5% (25/102)	3.2% (8/251)	10.8% (4/37)	17.3% (18/104)‡	4.3% (9/211)	9.1% (64/705)
p value	<0.01	<0.01		<0.01		

*Dashes indicate no isolates were drug resistant. †Intermediate resistance. With fractions, the numerator indicates the number of isolates with intermediate resistance, and the denominator indicates the total number of resistant isolates. ‡Numbers in column do not add up to 18 (the number of isolates) because of multidrug resistance.

In some isolates, antimicrobial drug resistance is tightly correlated with specific *emm* types (*13*). Resistance to erythromycin, clindamycin, and tetracycline is common in bacteria with the *emm*28 gene. In our study, 18/104 (17.3%) isolates that harbored *emm*28 were resistant to erythromycin, clindamycin, or tetracycline (p<0.01). Of all *emm* types, *emm*28 accounted for 71.0% (44/62) of all cases of resistance to these 3 antimicrobial drugs. In our study, 16/44 isolates harboring *emm*28 showed resistance to >2 antimicrobials. *emm*28 isolates in France were also found to be associated with multidrug resistance (*13*,*14*). Furthermore, in 2015, we found sharp increases in intermediate tetracycline resistance mainly in isolates harboring *emm*1 (57.9%, 22/38). Tetracycline resistance associated with *emm*12 and *emm*1 isolates was also found in scarlet fever patients in Hong Kong and China (*3*,*15*).

This study has a limitation. We collected samples from only 1 city in South Korea, the Gwangju metropolitan area. Because of the genetic diversity of GAS, our results should not be applied to other countries, even those nearby. However, we do believe that our data are representative of South Korea.

## Conclusions

In 2011, rapid increases in the incidence of scarlet fever in South Korea, as well as China and Hong Kong, reflected the beginning of a pandemic in Asia. However, the *emm* types contributing to disease differed from country to country. *emm*4, *emm*28, *emm*1, and *emm*3 were the most common *emm* types associated with scarlet fever in South Korea. Antimicrobial drug resistance in GAS in South Korea is closely associated with *emm*28, and resistance to tetracycline (observed emerging in 2015) is associated with type *emm*1. However, further studies are necessary to characterize the circulating strains and to control and prevent the further spread of scarlet fever.
